# Recovery of Cardiac Remodeling and Dysmetabolism by Pancreatic Islet Injury Improvement in Diabetic Rats after Yacon Leaf Extract Treatment

**DOI:** 10.1155/2018/1821359

**Published:** 2018-04-10

**Authors:** Klinsmann Carolo dos Santos, Sarah Santiloni Cury, Ana Paula Costa Rodrigues Ferraz, José Eduardo Corrente, Bianca Mariani Gonçalves, Luiz Henrique de Araújo Machado, Robson Francisco Carvalho, Ana Cláudia de Melo Stevanato Nakamune, Alexandre Todorovic Fabro, Paula Paccielli Freire, Camila Renata Corrêa

**Affiliations:** ^1^Medical School, São Paulo State University (UNESP), Botucatu, SP, Brazil; ^2^Institute of Biosciences, São Paulo State University (UNESP), Botucatu, SP, Brazil; ^3^School of Veterinary Medicine and Animal Science, São Paulo State University (UNESP), Botucatu, SP, Brazil; ^4^School of Dentistry, São Paulo State University (UNESP), Araçatuba, SP, Brazil; ^5^Medical School, University of São Paulo (USP), Ribeirão Preto, SP, Brazil

## Abstract

Yacon (*Smallanthus sonchifolius*) is a native Andean plant rich in phenolic compounds, and its effects on dysmetabolism and cardiomyopathy in diabetic rats was evaluated. The rats (10/group) were allocated as follows: C, controls; C + Y, controls treated with Yacon leaf extract (YLE); DM, diabetic controls; and DM + Y, diabetic rats treated with YLE. Type 1 diabetes (T1DM) was induced by the administration of streptozotocin (STZ; 40 mg^−1^/kg body weight, single dose, i.p.), and treated groups received 100 mg/kg body weight YLE daily via gavage for 30 d. The YLE group shows an improvement in dysmetabolism and cardiomyopathy in the diabetic condition (DM versus DM + Y) promoting a significant reduction of glycemia by 63.39%, an increase in insulin concentration by 49.30%, and a decrease in serum triacylglycerol and fatty acid contents by 0.39- and 0.43-fold, respectively, by ameliorating the pancreatic islet injury, as well as increasing the activity of the antioxidant enzymes (catalase, superoxide dismutase, and glutathione peroxidase) and decreasing the fibrosis and cellular disorganization in cardiac tissue. The apparent benefits of YLE seem to be mediated by ameliorating dysmetabolism and oxidative stress in pancreatic and cardiac tissues.

## 1. Introduction

Type 1 diabetes mellitus (T1DM) is a chronic state of insulin deficiency which results from the destruction of *β*-cells by the immune system, leading to a structural disorganization of the pancreatic islets. The Epidemiology of Diabetes Interventions and Complications study showed that intensive blood glucose control reduces the risk of several diseases, especially those related to the cardiac tissue [1].

Several studies have demonstrated that the development of cardiovascular disease is frequently observed in diabetic patients and in experimental models, and it is one of the major causes that elevates the incidence of morbidity [2–5]. Basically, in diabetic heart, there is a dramatic shift of the glucose utilization and almost complete reliance on fatty acid oxidation for energy production, resulting in the loss of metabolic flexibility as well as morphological changes in the cardiomyocytes [3, 6]. In summary, the dysmetabolism in diabetic heart leads to several biochemical and molecular pathway alterations [7]. Deregulated metabolism may be linked to increased production of reactive oxygen species (ROS) that leads to oxidative damage of DNA, proteins, and lipids as well as the activation of stress-sensitive pathways and development of cardiac oxidative stress in diabetes [8, 9].

The use of medicinal plants and herbs, for the treatment of many chronic diseases such as diabetes and its complications, has been recognized by a number of scientists and physicians based on their therapeutic properties [6]. Additionally, based on recommendations of the World Health Organization [10], antidiabetic agents derived from plants are an important alternative, as cotherapy, for the treatment of this condition. Over the past years, great attention has been given to the use of natural products, based on their pharmacological properties, as a form of complementary therapy.

Yacon (*Smallanthus sonchifolius*) is a perennial plant originally cultivated in the Andean highlands of South America, and its tubers are commonly used as food [11]. A number of studies have demonstrated the presence of large amounts of phenolic compounds in extracts from Yacon leaves and tubers [12, 13]. So, since diabetic cardiomyopathy is tightly related to oxidative stress—originally from persistent hyperglycemia and the metabolic shift in the cardiac tissue [14, 15], the treatment with Yacon leaves can be useful in diminishing oxidative damage and decreasing or preventing the progression of diabetic cardiomyopathy. Based on these information, the aim of the present study was to investigate the protective effect of Yacon leaf treatment on STZ-induced dysmetabolism and cardiomyopathy based on its antioxidant properties.

## 2. Material and Methods

### 2.1. Plant Material and Extract Preparation

The *S. sonchifolius* specimen was cultivated at the Department of Plant Science and Crop Protection, Federal University of Paraná, Curitiba, Paraná, Brazil. Briefly, the leaves from *S. sonchifolius* were dried for seven days at 50°C, powdered (3 *μ*m), and subjected to percolation at room temperature using a mixture of ethanol : H_2_O (7 : 3, *v*/*v*) with a flux of 2.0 mL/min/kg. The solvents were evaporated to dryness under low pressure (45°C) using a rotary evaporator in a vacuum system to afford the crude Yacon leaf extract (YLE). More information about the plant material, production of leaf extract, and phytochemical characterization can be found in a previous study of our group [13].

### 2.2. Experimental Design

**Figure 1:**
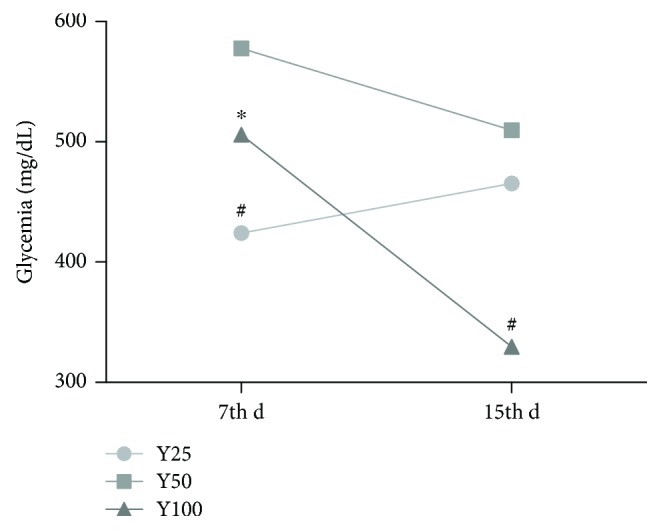
Dose-response profile of Yacon leaves. The animals were randomly assigned to one of three groups: Y25, Y50, and Y100 (25, 50, and 100 mg/kg body weight/day of Yacon extract constituted in 1 mL of 0.9% saline, resp.). Diabetes mellitus was induced by one i.p. administration of streptozotocin (STZ; 40 mg/body weight), and the animals received HEYL for gavage for 15 days after the establishment of the diabetic condition and glycemia was measured at day 7 (7th d) and day 15 (15th d). C: controls; C + Y: controls treated with Yacon leaf extract; DM: diabetic controls; and DM + Y: diabetic rats treated with Yacon. The data are represented as the median. Statistical analysis was performed using the generalized linear model and one-way analysis of variance test. Significant values are represented by *P* < 0.05. ^∗^Versus Y25; ^#^versus Y50.

Forty male Wistar rats, 60 d of age, were maintained in an environmentally controlled room (22 ± 3°C; 12-hour light/dark cycle and relative humidity of 60 ± 5%) and were fed with a standard rat pellet diet (Purina Ltd., Campinas, SP, Brazil) and water ad libitum. The experimental protocol was approved by the Ethics Committee on the Use of Animals (CEUA) at the Botucatu Medical School, São Paulo State University (UNESP) under number 1082-2014 (approved in April 24, 2014). The animals were randomly assigned to one of four groups (*n* = 10): control group (C); control group receiving YLE (C + Y); diabetic rats (DM); and diabetic rats receiving YLE (DM + Y). Diabetes mellitus was induced by the i.p. administration of streptozotocin for one time (STZ; 40 mg/kg body weight). Blood glucose was measured at 48 h and at 7 days after the STZ administration. The animals with blood glucose greater than 250 mg/dL were considered diabetic. The animals received YLE (100 mg/kg body weight/day constituted in 1 mL of 0.9% saline) for gavage for 30 days after the 7th day of the established diabetic condition. The dose of the treatment was selected based on a previous study conducted by our team [13], where 3 different doses were tested (Y25, Y50, and Y100) and the highest dose presented a better glycemic control (Figure 1). Control animals were given the same volume of saline. The animals were fasted overnight and killed by decapitation after anesthesia with ketamine (50 mg/kg body weight) and xylazine (0.5 mg/kg body weight) by intraperitoneal injection, and all efforts were made to minimize suffering. Blood was collected in tubes and then centrifuged at 3500 ×g. The serum and heart tissues were collected and stored at −80°C until analysis.

### 2.3. Biochemical and Hormonal Measurement

Serum glucose and triacylglycerol levels were measured using an automatic enzymatic analyzer system (biochemical analyzer BS-200, Mindray, China) and a commercial kit (Bioclin®, Belo Horizonte, Minas Gerais, Brazil), nonesterified fatty acid (NEFA) levels were determined by colorimetric kits (Wako NEFA-C, Wako Pure Chemical Industries, Tokyo, Japan), and insulin levels (EMD Millipore Corporation, Billerica, MA, USA) were measured by an immunoassay using a microplate reader (Spectra Max 190; Molecular Devices, Sunnyvale, CA, USA).

### 2.4. Redox State Markers

#### 2.4.1. Preparation of the Cardiac Tissue for Analysis

100 mg of the tissue was homogenized in 1.0 mL of a phosphate-buffered saline (PBS) pH 7.4 cold solution using a T 25 digital ULTRA-TURRAX® basic disperser (IKA® Werke Staufen, Germany) and centrifuged at 800*g* at 4°C for 10 min. The supernatant was used for measuring malondialdehyde and antioxidant enzyme activity levels, conducting histopathological analysis, immunohistochemistry, and fractal dimension analysis, and evaluating heart histology.

*(1) Malondialdehyde (MDA).* Briefly, we added 700 *μ*L of 1% orthophosphoric acid and 200 *μ*L of thiobarbituric acid (42 mM) to 100 *μ*L of the supernatant and then boiled it for 60 min in a water bath; the sample was cooled on ice immediately after that. 200 *μ*L was transferred to a 2 mL tube containing 200 *μ*L of sodium hydroxide-methanol (1 : 12 *v*/*v*). The sample was vortex mixed for 10 s and centrifuged for 3 min at 1.000 ×g. The supernatant (200 *μ*L) was transferred to a 300 *μ*L glass vial and 50 *μ*L was injected into the column. The HPLC was a Shimadzu LC-10AD system (Kyoto, Japan) equipped with a C18 Luna column (5 *μ*m, 150 × 4.60 mm, Phenomenex Inc., Torrance, CA, USA), a Shimadzu RF-535 fluorescence detector (excitation: 525 nm, emission: 551 nm), and 0.5 mL/min flow of phosphate buffer (KH_2_PO_4_ 1 mM, pH 6.8) [16]. MDA was quantified by area determination of the peaks in the chromatograms relative to a standard curve of known concentrations.

*(2) Antioxidant Enzymes.* Superoxide dismutase activity was measured based on the inhibition of a superoxide radical reaction with pyrogallol, and the absorbance values were measured at 420 nm [17]. Catalase activity was evaluated by following the decrease in the levels of hydrogen peroxide. The absorbance values were measured at 240 nm [18]. The activity is expressed as pmol of H_2_O_2_ reduced/min/mg protein. Glutathione peroxidase activity was measured by following *β*-nicotinamide adenine dinucleotide phosphate (NADPH) oxidation at 340 nm as described by Flohé and Günzler [19]; the results were expressed as *μ*mol of hydroperoxide reduced/min/mg protein. The values for the enzyme activities were corrected by protein content. Protein was quantified based on Lowry et al.'s method [20], using bovine serum albumin as the standard.

*(3) Pancreatic Histology and Pathologic Scoring.* For histopathological analysis, pancreatic tissue was fixed overnight in 10% formaldehyde, embedded in paraffin, and maintained in 70% ethanol until sliced. After being sliced in a microtome (4 *μ*m), cross sections were stained with hematoxylin and eosin (H&E). Pathology analyses were performed in pancreatic islets by scoring the tissue injury using a method described earlier with some modifications [21]. The total surface of the slide was scored for two different variables determining the severity of islet damage, such as the size and architecture of islets. The size criteria used were defined as follows: severe, less than 20% of the total field occupation; moderate, less than 50% of the total field occupation; slight, less than 70% of the total field occupation; and normal size islets, occupying around 70–80% of the field. For the architecture criteria, the following items were used: severe, islets presenting a nonsymmetric shape and totally disorganized nuclei; moderate, flat islets and clustered nuclei in the islet periphery; slight, semioval islets with 50% of the nuclei distributed more peripherally; and normal architecture islets, islets more rounded or oval and nuclei distributed symmetrically throughout the islet.

*(4) Immunohistochemistry.* The immunohistochemistry procedure was carried out following the manufacturer's protocol, starting with antigen retrieval for paraffin-sectioned slides using the standard laboratory protocol. The pancreas slides were incubated in the EnVision™ FLEX peroxidase-blocking reagent for 5 minutes to block endogenous enzyme activity. Subsequently, the EnVision FLEX anti-insulin primary antibody (1 : 2000) was incubated for 20 minutes, and after washing to remove primary antibodies, the slides were incubated with EnVision FLEX/horseradish peroxidase (HRP) for 20 minutes, followed by two washes, and the procedure continued by incubating the slides with diaminobenzidene (DAB) chromogen for 10 minutes. Finally, the slides were stained with hematoxylin and dried for xylene preparation. Insulin was quantified through ImageJ® image processing program.

*(5) Heart Histology.* Hearts were harvested for histological evaluation of potential fibrosis and tissue disorganization. The hearts were fixed in 10% formaldehyde overnight, embedded in paraffin, and maintained in 70% ethanol until sliced. The hearts were sliced, with cross sections about 4 *μ*m thick, using a microtome. The staining with H&E and Picro Sirius Red (PSR) was performed according to standard histological processing. PSR stained sections were used to quantify the collagen area using the ImageJ image processing program, following software instructions. The fractal dimension was accessed using H&E stained sections. Three random sections from each animal were photographed through a 20x objective lens, using a light microscope (Leica, Germany).

*(6) Fractal Dimension Analysis.* To quantify the heart nucleus disorganization, H&E stained sections were analyzed using the fractal dimension methodology based on Pacagnelli et al.'s description [22]. Using the ImageJ software, the images of the slides with the heart tissue were binarized, and the fractal dimension was estimated by using a tool that quantifies pixel distribution in the binarized images; this tool, “Fractal box-count,” is capable of generating a fractal dimension value (*D*), which ranges from 0 to 2. A value close to 2 represents more pixel disorganization.

### 2.5. Statistical Analysis

For normally distributed data, the analyses were performed using two-way ANOVA followed by the nonparametric test. For scoring islet damage, the linear model for a binomial distribution with a logistic link was used, followed by the multiple Wald comparison test for islet size and architecture. Statistical tests were performed using SAS for Windows, v. 9.3. Statistical significance was considered when *P* < 0.05.

## 3. Results

### 3.1. Yacon Treatment Improves Dysmetabolism in Diabetic Condition

After 30 d of treatment with 100 mg/kg body weight/d—based on a dose-response pilot study (Figure 1), YLE promoted a significant reduction of glycemia at 63.39% in the DM + Y group when compared to the untreated group, whereas it increased the insulin concentration by 49.30% in the treated group (Table 1). Additionally, Yacon treatment decreased the serum TG and NEFA contents (DM versus DM + Y) by 0.39- and 0.43-fold, respectively (Table 1). On the other hand, treatment with Yacon increased the TG content in the control group when compared to the untreated control group.

### 3.2. Yacon Treatment Ameliorates Oxidative Stress Markers in Heart Tissue

**Figure 2:**
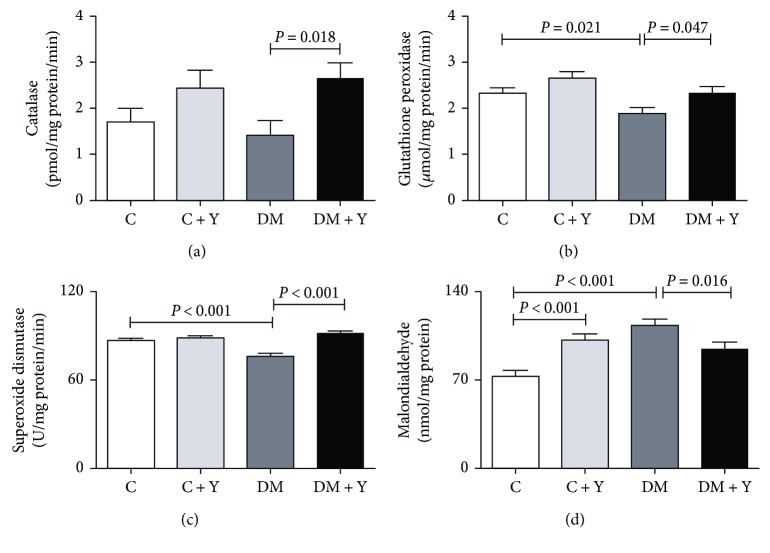
Redox state markers. (a) Catalase; (b) glutathione peroxidase; (c) superoxide dismutase activities; and (d) malondialdehyde concentration in the cardiac tissue. The data are represented as the mean ± SEM. Significance were represented by *P* values. C: controls; C + Y: controls treated with Yacon leaf extract; DM: diabetic controls; DM + Y: diabetic rats treated with Yacon.

To investigate the effect of Yacon treatment in redox status response in the heart of diabetic animals, markers of antioxidant defense and oxidative stress were measured. The antioxidant defense enzymes catalase, glutathione peroxidase, and superoxide dismutase levels were significantly decreased in the DM group compared with the control group, and increased in diabetic animals treated with Yacon compared to untreated diabetic animals (Figures 2(a)–2(c)). In an opposite manner, the oxidative stress marker (malondialdehyde) level was increased in the DM group compared to the control group and decreased in diabetic animals treated with Yacon when compared with the DM group. We also verified an increase in this marker in the control group treated with Yacon when compared to the untreated control group (Figure 2(d)).

### 3.3. Yacon Treatment Reduces Pancreas Severe Phenotype and Increases Insulin Production

**Figure 3:**
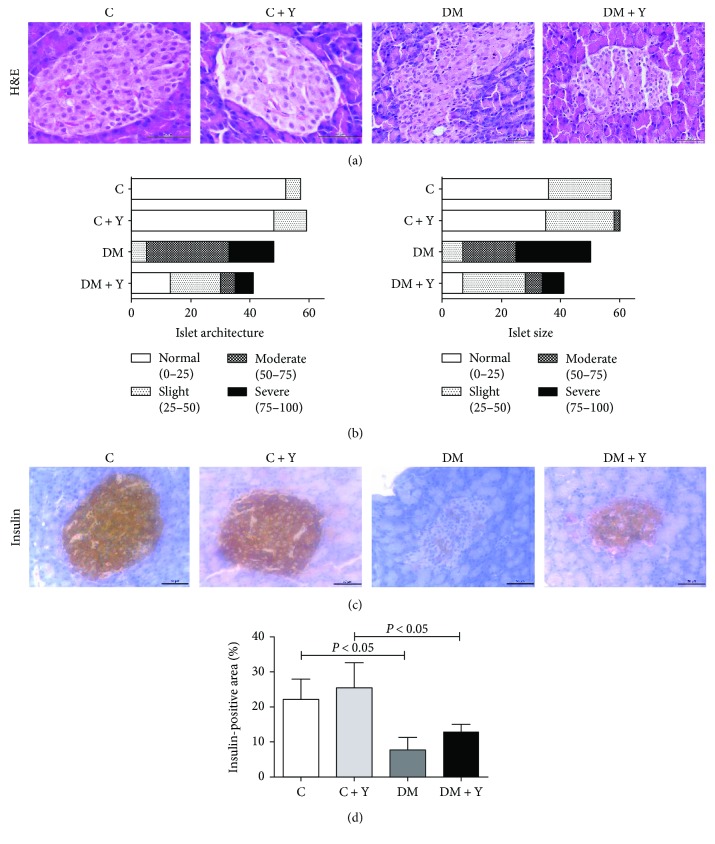
Pancreatic histology, pathologic scoring, and immunohistochemistry analysis. (a) Pancreas histological sections stained with hematoxylin and eosin. (b) Pancreas scoring among groups using architecture and islet size as parameters. (c) Histological analysis of the pancreas by immunohistochemistry for insulin. (d) Quantitative analysis of the positive area for insulin in the pancreatic islets quantified using the imaging software ImageJ. Original magnification, ×20. Scale bars, 50 *μ*m. The data represent the mean ± standard deviation. Statistical analysis was performed using the generalized linear model and two-way analysis of variance test complemented with a nonparametric test for the insulin-positive area or the generalized linear model (binomial) followed by the Wald test for scoring analysis. Significance were represented by *P* values. C: controls; C + Y: controls treated with Yacon leaf extract; DM: diabetic controls; DM + Y: diabetic rats treated with Yacon; H&E: hematoxylin and eosin.

To analyze the effect of the treatment on the Langerhans islet size and architecture, we used a linear model for binomial distribution with a logistic link followed by the multiple comparison Wald test. For the analysis of the islet number, the results showed that the DM group presented fewer islets with a normal size compared to the control group, and the treatment with Yacon (DM + Y) increased the number of islets with a normal size (Figure 3(b)). For the slight, moderate, and severe alterations, the DM group showed an increase in islets with these characteristics, whereas the treatment seems to prevent these alterations in the diabetic group (Figure 3(b)). Moreover, the architecture analysis showed a reduction in the number of islets with a normal architecture. Notably, we verified a higher number of islets with an altered architecture (moderate and severe) in DM when compared to DM + Y, suggesting that the treatment with Yacon could alleviate the islet architecture deterioration in the diabetic condition. No moderate or severe changes were observed in the control group (Figure 3(b)). The description of the results for the islet staging criteria are exposed in Table S1 and Figure S1 (Supplementary Materials).

### 3.4. Yacon Decreases Fibrosis and Nuclear Disorganization in the Heart of Diabetic Rats

**Figure 4:**
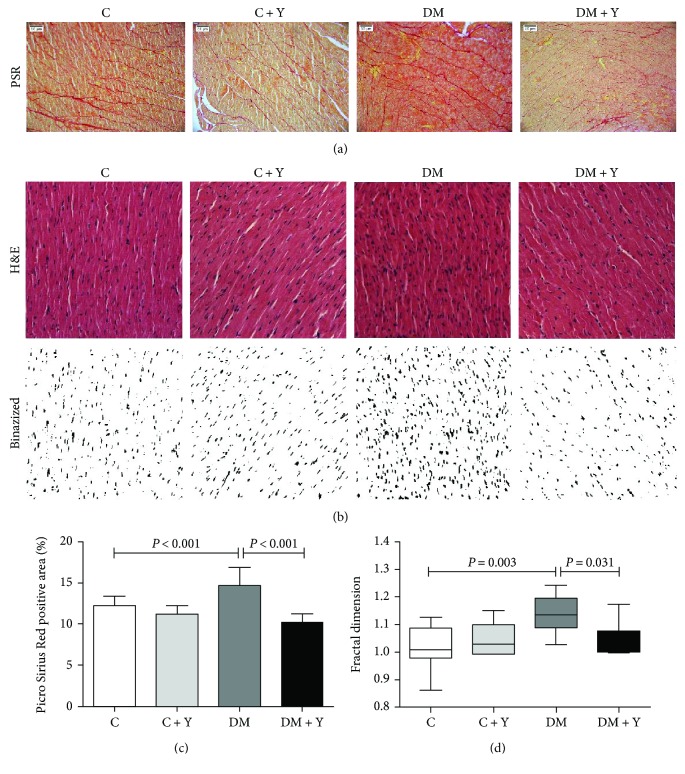
Extracellular matrix collagen area and nuclear disorganization in the cardiac tissue. (a) Heart histological sections of control, diabetic, and treated animals stained with Picro Sirius Red to access extracellular matrix fibrosis. (b) Histological sections of the heart stained with hematoxylin and eosin and the corresponding images after binarization of each group. Original magnification, ×20. Scale bars, 50 *μ*m. (c) Quantitative analysis of the collagen area in the PSR-stained sections. (d) Fractal dimension quantified by imaging software ImageJ. The data represent the mean ± standard deviation, and for fractal analysis data are expressed as a box plot graphic showing the first and third quartiles and the median, minimum, and maximum. Statistical analysis was performed using the two-way analysis of variance test complemented with a nonparametric test. Significance were represented by *P* values. C: controls; C + Y: controls treated with Yacon leaf extract; DM: diabetic controls; and DM + Y: diabetic rats treated with Yacon; PSR: Picro Sirius Red; H&E: hematoxylin and eosin.

To verify the effect of the treatment in diabetic heart fibrosis, we analyzed histological sections stained with PSR, which is responsible for collagen staining. Animals belonging to the untreated diabetic group present an increased collagen area in the extracellular matrix compared to that of the control group. Furthermore, the diabetic rats treated with Yacon showed a decrease in fibrosis accumulation compared to the rats of the DM group (Figures 4(a) and 4(c)). Moreover, we analyzed heart tissue organization by assessing the fractal dimension of nucleus localization using histological sections stained with H&E. Fractal analysis showed that the nucleus disorganization in diabetics is worse than that in the control. Additionally, the treatment with Yacon was able to reverse the nuclear disorganization in diabetics (Figures 4(b) and 4(d)). These data indicate the beneficial effect of Yacon in reducing heart extracellular matrix fibrosis in the diabetic condition and the potential to ameliorate heart tissue disorganization.

## 4. Discussion

It is known that the hyperglycemia that occurs in diabetes is a major cause of diabetic complications. Since the STZ model of diabetes induction causes the destruction of pancreatic *β*-cells, simulating the physiopathological process of the disease, it generates a deficiency in insulin biosynthesis and secretion and, consequently, an increase in serum glycemia content since it becomes unavailable to insulin-sensitive tissue. In the present study, diabetic animals underwent a hyperglycemic condition after 7 d of STZ administration (Table 1). This condition can be better visualized based on the histological analyses where the DM group presented a deterioration of the architecture and size of the islets (Figures 3(a) and 3(b)) and a decrease in insulin production (Figures 3(c) and 3(d)). Additionally, under these conditions, the breakdown of the structural protein and lipolysis is increased, promoting weight loss and an increase of circulating lipids (e.g., triacylglycerol and fatty acid) [3]. Concomitantly, the serum levels of NEFA were enhanced in the DM group (Table 1); this represents an increase in the availability of NEFA to the heart for energy generation since the cardiac tissue (insulin dependent) does not utilize glucose adequately as an energy source under a diabetic condition [23]. An increase in myocardial fatty acid uptake and oxidation has been described in humans with both type 1 and type 2 diabetes, as well as in many animal models [24]. Elevated circulating glucose [25] and free fatty acid [26, 27] have been shown to possess an important role in the complications of both type 1 and type 2 diabetes. On the other hand, the treated diabetic group presented a decrease in serum glucose and circulating lipids. This can be attributed to an improvement of the glycemic control, suggesting a greater peripheral utilization of glucose and the maintenance of the adipose and muscle tissues. These biochemical and hormonal improvements are potentially linked to the preservation and/or regeneration of the remaining pancreatic islets that were partially destroyed by STZ and, consequently, the potentiation of the insulin secretion from the protected/regenerated *β*-cells [28, 29]. In agreement with our data, some authors demonstrated that the hydroethanolic extract of Yacon leaves significantly reduced glucose levels in diabetic rats and in genetically type 2 diabetic mice [11, 30, 31]; in parallel, Honoré et al. [32] and Habib et al. [33], respectively, demonstrated that immunofluorescent staining of the pancreatic tissues in a diabetic animal presented a decrease of insulin density whereas diabetic rats treated with a Yacon leaf decoction or Yacon root flour showed strong insulin immunostaining.

In summary, there is extensive evidence that the hyperglycemia established in the diabetic condition is associated with the generation of reactive oxygen species (ROS) and a weakening of the antioxidant defense, resulting in enhanced oxidative stress [13, 34]. Indeed, the activity of several antioxidant enzymes is decreased in the diabetic heart in both rats and humans [13, 35, 36]. Here, we have demonstrated that the activity of the antioxidant enzymes catalase, superoxide dismutase, and glutathione peroxidase is decreased in the diabetic group and MDA concentration is elevated in cardiac tissue (Figures 2(a)–2(d)), indicating an oxidative stress process. These enzymes are regarded as the first line of the antioxidant defense system and work together to diminish ROS generation during oxidative stress [37]. The deleterious effects of oxidative stress in the diabetic heart are well established, including cell death and cardiac fibrosis [38–40]. Cardiac fibrosis is a major feature of diabetic cardiomyopathy [41]. Diabetic cardiomyopathy is defined as a ventricular dysfunction that occurs in diabetic patients not related to another cause (e.g., hypertension or coronary artery disease) [4, 42]. Somaratne et al. [43] reported that 56% of diabetic patients had diabetic cardiomyopathy. Although the etiology of the diabetic cardiomyopathy is not yet completely understood, the pathophysiology of this condition is believed to be multifactorial. Existing evidence suggests that persistent hyperglycemia-induced oxidative stress is an important contributor to it [14, 15]. In this condition, an excessive production of the extracellular matrix protein leads to an increased myocardial hardness and consequent cardiac dysfunction and, consequently, resulting in cardiac failure [44]. Concomitantly, our data indicate that the diabetic condition leads to an increase of collagen deposit and nuclear disorganization in cardiac tissue (Figures 4(a)–4(d)). Diabetic patients have a 2- to 5-fold increased risk of developing heart failure, one of the greatest contributors to morbidity and mortality [45]. Here we have also showed an increase of oxidative stress in the heart of the untreated DM group, whereas the Yacon treatment promoted a decrease of the oxidative stress marker and an increase of the antioxidant enzyme activity. We have previously demonstrated the antioxidant activity of Yacon leaves in the soleus muscle, potentially due to the presence of several antioxidant compounds in the extract [13]. Additionally, phytochemical studies of Yacon leaves showed the presence of high-polarity antioxidant compounds such as caffeic, chlorogenic, and three dicaffeoylquinic acids [46]. It is known that compounds such as phenolic acids, polyphenols, and flavonoids can scavenge free radicals such as peroxide, hydroperoxide, or lipid peroxyl and thus inhibit the oxidative mechanisms that lead to diabetes complications. So, since oxidative stress is related to the cardiac remodeling and fibrosis through several mechanisms [38, 39, 47], this could be the mechanism by which DM + Y presented an improvement of the cardiac alterations mentioned above. Given the fundamental role of oxidative stress in the pathogenesis of diabetes and diabetic cardiomyopathy, there is growing interest in the use of antioxidants as a complementary therapeutic approach to prevent/treat these conditions. Numerous studies demonstrated that ameliorating oxidative stress through antioxidant treatment might be an effective strategy for reducing diabetic cardiomyopathy [48, 49].

The important findings of this study are the cardio- and pancreatic protective effects of Yacon treatment in experimental STZ-induced diabetic cardiomyopathy and pancreatic islet dysfunction in terms of the preservation of the Langerhans islet architecture and insulin production as well as an inhibition of collagen content accumulation and enhancement of antioxidant enzyme activities in cardiac tissue. Summarizing, our results demonstrated that STZ administration successfully induced diabetes and diabetic cardiomyopathy as indicated by the fractal analysis, indicating cellular disorganization, as well as an increase in collagen deposition in heart tissue and a decrease of insulin production and preservation of the architecture of the pancreatic *β*-cells. Interestingly, these cardiac and pancreatic abnormalities were improved by the administration of the Yacon extract. Furthermore, exploring the potential therapeutic effects of plants and herbal medicine could contribute to the detection of new targets and treatments. Our findings, even as a basic research model, provide information that can guide future studies aimed at elucidating new therapeutic alternatives for diabetic complications.

## Figures and Tables

**Table 1:** Serum biochemical and hormonal outcomes.

Outcomes	Groups			
	C	C + Y	DM	DM + Y
Initial glycemia (mg/dL)	128 ± 14.75	119.6 ± 14.76	416.11 ± 15.55^∗^	395.33 ± 19.05^#^
Final glycemia (mg/dL)	94 ± 17.90	88.66 ± 18.87	299.75 ± 20.02^∗^	109.71 ± 19.46^†^
Insulin (pmol/L)	32.25 ± 2.45	30.87 ± 2.60	20.24 ± 3.30^∗^	30.22 ± 3.01^†^
TG (mg/dL)	54.74 ± 3.26	71.53 ± 3.64^∗^	89.77 ± 3.89^∗^	64.42 ± 4.21^†^
NEFA (mEq/L)	0.34 ± 0.03	0.38 ± 0.03	0.43 ± 0.03	0.30 ± 0.03^†^

The data are represented as the mean ± SEM. Statistical analysis was performed using the generalized linear model and two-way analysis of variance test complemented with a nonparametric test. Significant values are represented by *P* < 0.05. ^∗^Versus C; ^#^versus C + Y; ^†^versus DM; C: controls; C + Y: controls treated with Yacon leaf extract; DM: diabetic controls; DM + Y: diabetic rats treated with Yacon; TG: triacylglycerol; NEFA: nonesterified fatty acid.

## Supplementary Materials

Supplementary Materials Table S1: analysis of the islets by scores. Description of the results for the islet staging criteria (normal, slight, moderate, and severe) obtained after the linear model for a binomial distribution with a logistic link followed by the multiple Wald comparison test for islet size and architecture. C: controls; C + Y: controls treated with Yacon leaf extract; DM: diabetic controls; and DM + Y: diabetic rats treated with Yacon; ND: no difference; NT: not tested. The linear model for a binomial distribution followed by the multiple Wald comparison test analyzed statistical significance. Figure S1: analysis of the islets by scores. Description of the results for the islet staging criteria (normal, slight, moderate, and severe) obtained after the linear model for a binomial distribution with a logistic link followed by the multiple Wald comparison test for islet size and architecture. Blue lines represent statistical significance while red lines mean no difference (ND) or not tested (NT) among the groups.
