# Supplementary material for: Recovery of Cardiac Remodeling and Dysmetabolism by Pancreatic Islet Injury Improvement in Diabetic Rats after Yacon Leaf Extract Treatment
Source: Oxid Med Cell Longev. 2018 Apr 10;2018:1821359. doi: 10.1155/2018/1821359 (PMC6051012; doi:10.1155/2018/1821359)
Supplement: Supplementary Materials — Table S1: analysis of the islets by scores. Description of the results for the islet staging criteria (normal, slight, moderate, and severe) obtained after the linear model for a binomial distribution with a logistic link followed by the multiple Wald comparison test for islet size and architecture. C: controls; C + Y: controls treated with Yacon leaf extract; DM: diabetic controls; and DM + Y: diabetic rats treated with Yacon; ND: no difference; NT: not tested. The linear model for a binomial distribution followed by the multiple Wald comparison test analyzed statistical significance. Figure S1: analysis of the islets by scores. Description of the results for the islet staging criteria (normal, slight, moderate, and severe) obtained after the linear model for a binomial distribution with a logistic link followed by the multiple Wald comparison test for islet size and architecture. Blue lines represent statistical significance while red lines mean no difference (ND) or not tested (NT) among the groups. [file 1821359.f1.pdf]

Supplementary Material

Table S1. Analysis of the islets by scores. Description of the results for the islets staging criteria (normal, slight, moderate and severe) obtained after the linear model for a binomial distribution with logistic link followed by multiple Wald comparison test for islet size and architecture.

		C vs. DM	C vs. C+Y	C+Y vs. DM+Y	DM vs. DM+Y
**Size**	**Normal**	P < 0.001*	ND or NT	P < 0.001*	P < 0.001*
	**Slight**	P = 0.0094*	ND or NT	ND or NT	P < 0.001*
	**Moderate**	P < 0.001*	ND or NT	ND or NT	P = 0.025*
	**Severe**	ND or NT	ND or NT	ND or NT	P = 0.0017*
**Architecture**	**Normal**	P < 0.001*	ND or NT	P < 0.001*	P < 0.001*
	**Slight**	ND or NT	ND or NT	ND or NT	P = 0.004*
	**Moderate**	ND or NT	ND or NT	ND or NT	P < 0.001*
	**Severe**	ND or NT	ND or NT	ND or NT	ND or NT

C: Control; C+Y: Control treated with Yacon; DM: Diabetes Mellitus; DM+Y: Diabetes Mellitus treated with Yacon; ND: No difference; NT: Not tested. The linear model for a binomial distribution followed by multiple Wald comparison test analyzed statistical significance.

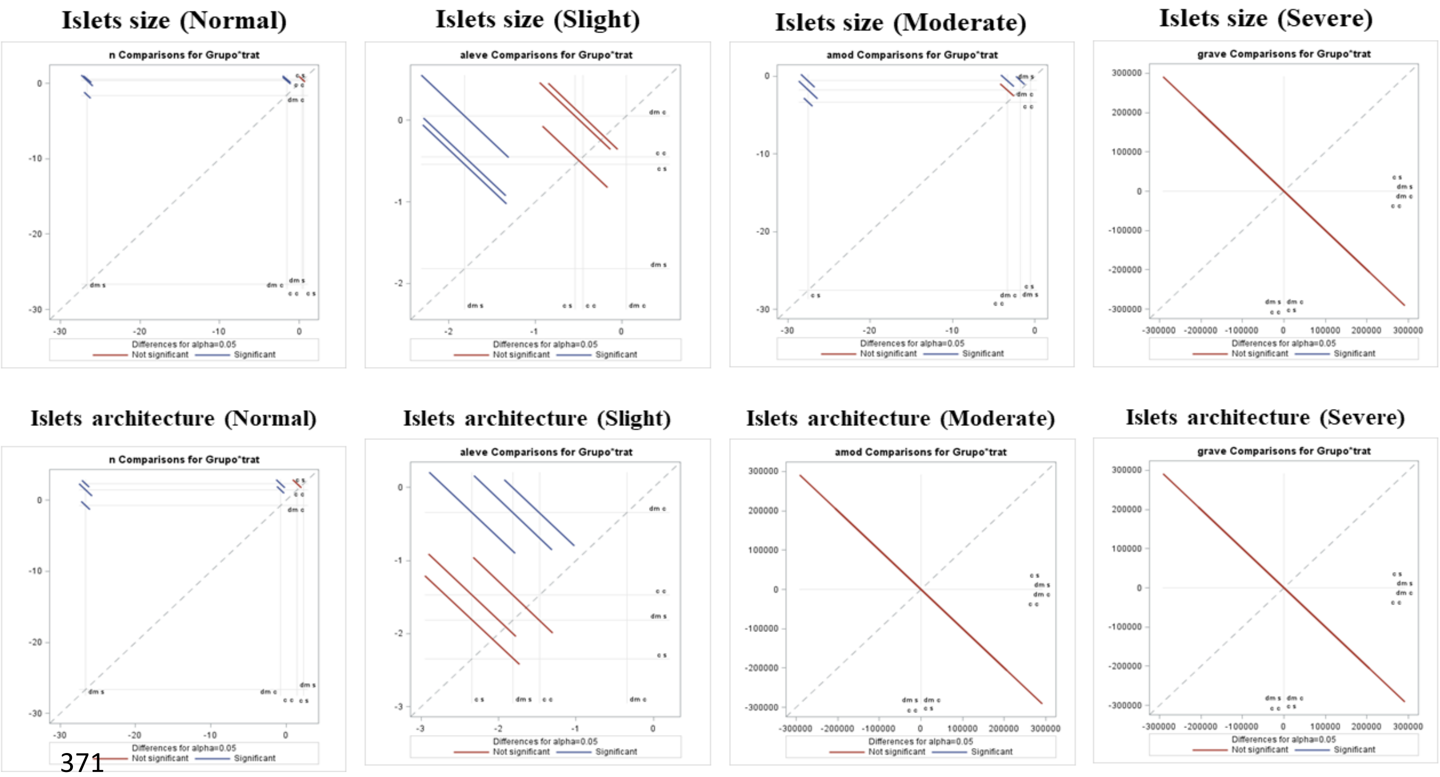

Figure S1. Analysis of the islets by scores. Description of the results for the islets staging criteria (normal, slight, moderate and severe) obtained after the linear model for a binomial distribution with logistic link followed by multiple Wald comparison test for islet size and architecture. Blue lines represent statistical significance while red lines mean no difference (ND) or not tested (NT) among the groups.
